# Influence of an Implant Fixture including a Freely Removable Micro-Locking Implant Prosthesis on Peri-Implant Tissues and Implant Prostheses: A Prospective Clinical Study

**DOI:** 10.3390/jcm10153321

**Published:** 2021-07-28

**Authors:** Young-Gun Shin, Won-Tak Cho, Ho-Kyung Lim, Su-Hyun Hwang, Ji-Hyeon Bae, Gang-Ho Bae, Jeong-Yol Lee, Jung-Bo Huh

**Affiliations:** 1 Department of Prosthodontics, Dental Research Institute, Dental and Life Sciences Institute, Education and Research Team for Life Science on Dentistry, School of Dentistry, Pusan National University, Yangsan 50612, Korea; sinyg99@naver.com (Y.-G.S.); joonetak@hanmail.net (W.-T.C.); hsh2942@hanmail.net (S.-H.H.); say0739@daum.net (J.-H.B.); qorkdgh97@naver.com (G.-H.B.); 2 Department of Oral and Maxillofacial Surgery, Korea University Guro Hospital, Seoul 08308, Korea; ungassi@naver.com; 3 Department of Prosthodontics, Korea University Guro Hospital, Seoul 08308, Korea

**Keywords:** micro-locking, dental implant, fixture, abutment, prospective study, survival rate, complications

## Abstract

This prospective study was undertaken to evaluate the clinical usefulness of a newly developed one-piece, screw-free, and micro-locking implant system, which was designed to overcome the shortcomings of the existing implant systems. Thirty-eight patients were recruited and randomly and equally assigned to an experimental group (micro-locking one-piece fixture, MLF; *n* = 19) or a control group (micro-locking abutment, MLA). Cumulative implant survival rates, marginal bone resorptions, probing depths, plaque indices, bleeding indices, and complications were obtained by using clinical and radiographic findings at 6 months and 12 months after prosthesis placement. Complications that occurred multiple times for single implants were counted. During the 12 month observation period, survival rates were 100% in both groups. No significant intergroup differences were observed for marginal bone resorption, probe depth, or bleeding index. However, mean plaque index was significantly lower in the MLF group at 12 months (*p* < 0.05). During the 12-month observation period, food impaction (26.3%) was the main complication in the MLF group and screw loosening (5.3%), prosthesis detachment (5.3%), and food impaction (5.3%) were observed in the MLA group. The results of this study suggest that the one-piece micro-locking implant system offers a predictable treatment method.

## 1. Introduction

Implant-supported fixed dental prostheses function similar to natural teeth, but unlike natural teeth they consist of a fixture, abutment, and upper prosthesis [1,2,3]. Implant-supported fixed dental prostheses are classified as screw-retained or cement-retained according to the methods used to retain the upper prosthesis. Screw-retained prostheses achieve stability and retention due to the clamping force exerted by screw tightening, whereas cement-retained prostheses achieve stability due to adhesive effects [2,3]. Both implant-supported fixed prosthesis types have high success rates. However, they have relative advantages and disadvantages, which can affect the incidences of biomechanical complications [2,3,4,5].

Screw-retained prostheses are easily removed and repair and hygiene management are straightforward and free of complications such as peri-implantitis, swelling, and ulcers caused by residual cement surrounding the implant prostheses and abutments [6,7,8]. However, screw-retained prostheses require a high degree of precision to ensure a passive fit [9,10] and possess mechanical complications such as screw loosening and fractures [3,11]. In addition, screw-retained prostheses have a hole for screw access [7,12,13] and this interferes with natural occlusion and the continuity of porcelain veneer on occlusal surfaces [7,14,15,16].

On the other hand, a passive fit can be easily achieved by using cement-retained prostheses because the cement layer compensates for errors in the manufacturing process and transmits stress uniformly to prostheses, implants, and the alveolar bone [6,15,17,18]. In addition, since cement-retained prostheses do not possess a screw access hole, aesthetics and proper occlusion are easily achieved [14,17,19]. Furthermore, the laboratory processing of cement-retained prostheses is similar to that which is required for tooth-retained prostheses [19]. However, retention control is an issue for cement-retained prostheses and difficulties may be experienced in achieving appropriate retention when the intermaxillary space is insufficient or in retrieving the prostheses for repair and maintenance [5,6,19,20]. In addition, subgingival cement removal may damage the abutment and residual cement may cause biological complications such as peri-implantitis [6,19,20,21].

In order to address the problems associated with screw and cement-retained prostheses, a screw and cement-retained prosthesis (SCRP) was developed that combines the advantages of these prostheses types [9,22,23]. SCRPs are cement-retained prostheses with a screw hole on the occlusal surface and a specially designed abutment [9]; these can achieve passive fit and reduce prosthesis detachment associated with the use of permanent cement [10,24]. The abutment and prosthesis are permanently cemented and can be removed from the fixture. Moreover, the residual cement can be completely removed from outside the oral cavity [24]. Therefore, it is possible to reduce abutment scratches that may occur when cement is removed after prosthesis cementation and to minimize the risk of peri-implantitis due to residual cement [8,21]. However, this method also requires a screw hole in the occlusal surface [9,24].

Recently, a new type of implant prosthesis retention system was introduced by Samwon DMP (Yangsan, Korea) in order to overcome the shortcomings of existing implant prostheses and allow operators to easily attach and detach prostheses when desired [25]. The screw-integrated abutment is tightened to the installed fixture and then a ready-made cylinder is mounted above on the abutment and an upper prosthesis is attached to this cylinder by resin cementation. A spring made of nickel-titanium shaped memory alloy applies a continuous and constant force on the zirconia ball inside the cylinder placed in the retention groove of an abutment. The micro-locking abutment (the MLA system, Samwon DMP, Yangsan, Korea) is described as a freely removable system because it can achieve retention without a screw or cement between the final prosthesis and implant abutment [26]. Furthermore, this system is aesthetic and advantageous for forming occlusal points because no screw access hole is required. When it is difficult to remove an MLA prosthesis due to strong retention, it can be easily removed by using a dedicated driver by forming a hole, which can be smaller than the existing screw access hole. Since this implant fixture is freely removable, it is possible to remove excess cement from outside the mouth, which can prevent biological complications such as peri-implantitis and has considerable maintenance advantages [18,27,28].

An in vitro pilot study on the retention of the MLA system showed it has a low retention loss rate at 12 months and provides stable retention thereafter [25]. In addition, several case reports and retrospective clinical studies have reported successful clinical applications of the system. Previous case reports and clinical studies used a two-piece ML system incorporated into the abutment. The MLA system is connected to fixtures by using an abutment screw, such as the conventional screw-retained implant prostheses, and this connection method is easily applied to existing implant systems. However, complications such as the screw loosening or screw fractures are possible [25,26,27,28,29].

The one-piece micro-locking implant system (the MLF system), which was designed to prevent mechanical complications, combines fixture and abutment and, thus, screw-related mechanical complications are circumvented by the lack of a screw component [30]. In order to exclude screw-related mechanical complications, an implant fixture including an ML system that is similar to a one-piece implant was developed. To date, few clinical studies have been conducted on implant fixtures such as the MLA or MLF systems [25,26,27,28,29] and, in order to establish a scientific basis, long-term clinical trials are required for the one-piece ML system. In the present study, marginal bone loss and complications were evaluated by prospective clinical examination and radiological analysis in patients fitted with a screw-free one-piece MLF or a two-piece MLA system. The purpose of this study was to evaluate and compare the clinical usefulness and complications associated with the newly developed one-piece MLF and a two-piece MLA.

## 2. Materials and Methods

### 2.1. Research Subjects

This study was conducted from January 2018 to May 2020 in the Department of Prosthodontics at Pusan National University Dental Hospital and Korea University Dental Hospital on patients that required implant prosthetic treatment due to tooth loss. Patients that participated in the study received explanation of the purpose and contents of the study and volunteered to participate. Patients with systemic factors (such as taking bisphosphonate drugs) that might have affected prosthesis prognoses, patients receiving chemotherapy, patients with a systemic disease that might directly affect prognosis, and patients without a follow-up check were excluded. A total of 38 patients that met these inclusion criteria were randomly assigned (using a random number table) to an MLF group (micro-locking one-piece fixture; *n* = 19) in which the one-piece implant fixture included the ML system and the other 19 patients were assigned by block randomization to a MLA group (micro-locking abutment; *n* = 19) by a clinical research coordinator. Since fixtures were delivered just before implant placement, the operator checked the fixture structures and was not blinded. Table 1 shows the distributions of subject in the two experimental groups. The study was approved beforehand by the Institutional Review Board of Pusan National University Dental Hospital (IRB No. PNUDH-2018-003-MD).

### 2.2. Micro-Locking Implant Prosthesis (ML) System

**Figure 1:**
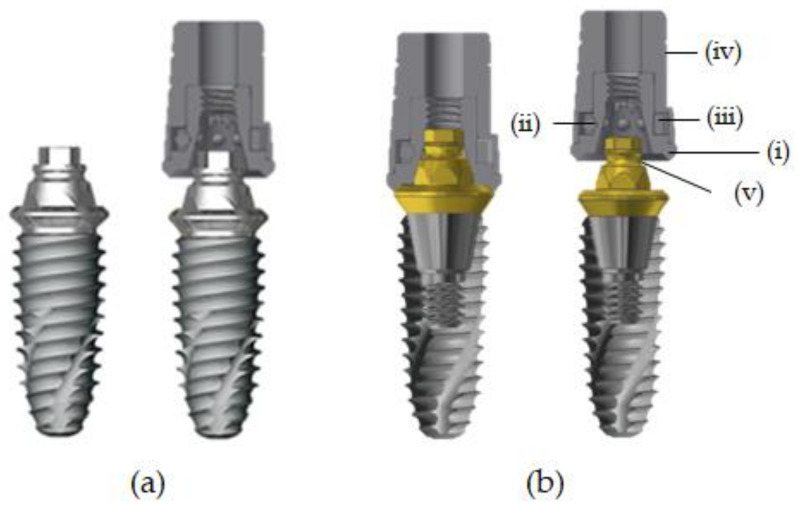
Cross section of both groups. (**a**) MLF (micro-locking one-piece fixture), (**b**) MLA (micro-locking abutment), (**i**) body, (**ii**) ball, (**iii**) spring, (**iv**) cap, and (**v**) retention groove.

**Figure 2:**
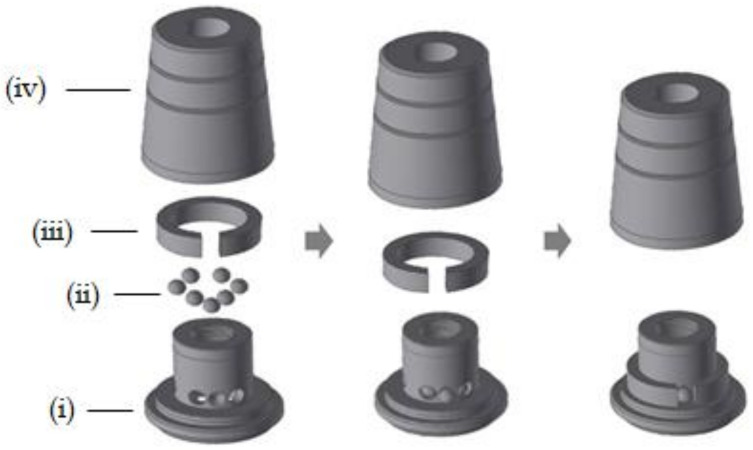
Illustration of the cap assembly process. (**i**) Body, (**ii**) ball, (**iii**) spring, and (**iv**) cap.

The ML system used in this study was included in the fixture in the MLF group and the abutment in the MLA group. The upper part of the ML system is composed of a cap and the two groups had an upper cap of the same shape. The cap consisted of four sub-elements: body, ball, spring, and cap. The body had a hexagonal receptacle with multiple grooves that matched the hexagonal structure of the abutment cylinder to prevent prosthesis rotation (Figure 1 and Figure 2).

The ball was composed of zirconium oxide (ZrO_2_) and hafnium oxide (HfO_2_) and the spring, which surrounds the zirconia ball, is composed of a nickel-titanium (Ni-Ti) shape memory alloy. The nickel-titanium spring is located inside the cap and, thus, the spring is not in direct contact with the surrounding tissue. Nickel is known to be a causative agent for allergies, but the literature reports that allergic reactions in Ni-Ti alloys are extremely rare [31]. The Ni-Ti alloy has excellent corrosion resistance and biocompatibility and is used for the manufacture of catheters and cardiovascular stents [32]. This structure maintains constant stress and component retention is restored even after large strain distortions. Therefore, the prosthesis can be attached and detached without structural deformation or loss of retention. The spring used in this study expands slightly when the cap is engaged with the abutment, which allows the cap to be easily positioned on the undercut of the retention groove. When the prosthesis and implant are combined, a constant external force is applied to the ball (Figure 1) [25].

**Figure 3:**
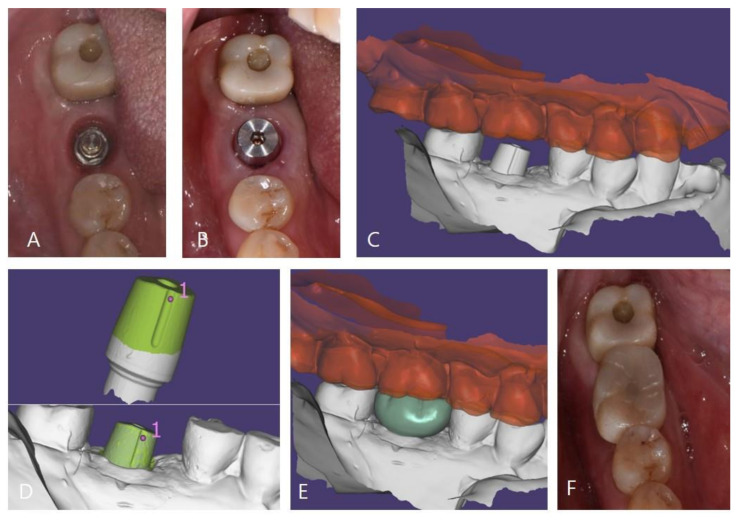
Prosthesis setting process of the ML system. (**A**): Exposed connection of the ML system, (**B**): attached cap, (**C**): model scanned data, (**D**): superimposing pre-scanned cap data onto model scanned data, (**E**): designed zirconia crown, and (**F**): zirconia crown on the cap fixed with resin cement.

In the MLA group, the abutment was tightened to 35 N/cm according to the manufacturer’s instructions before taking an impression for the prosthesis and then the cap was attached to the abutment by using a dedicated tool. In the MLF group, the ML system was included in the fixture and, thus, the cap was directly attached to the implant. The subsequent impression-taking process was identical in both groups. The impressions were obtained by using silicone impression material (Imprint II VPS, 3M ESPE, Seefeld, Germany) and a fabricated master cast was scanned by using a 3D scanner (Trios 3, 3Shape, Copenhagen, Denmark). Zirconia crowns were manufactured by using a CAD-CAM system (Exocad DentalCAD, Exo-cad GmbH, Darmstadt, Germany) (Trione Z, Dio Implants, Pusan, Korea). After evaluating marginal suitability, esthetics, and occlusion in the oral cavity, the crowns were cemented to caps by using self-adhesive resin cement (G-CEM LinkAce; GC, Tokyo, Japan). Cemented crowns and caps were then removed by using a dedicated removal driver, excess cement was removed, and the crown margins were polished. Crowns cemented to caps were then reattached to abutments and access holes on occlusal surfaces were filled with a packable composite resin (Filtek Z350XT, 3M ESPE, St. Paul, MN, USA) (Figure 3) [26,33].

### 2.3. Clinical Examination

The checkup items detailed below were evaluated by clinical examination and by using radiographs during regular follow-up checks at 6 months and 12 months after prosthesis placement.

#### 2.3.1. Cumulative Implant Survival Rates

The main outcomes of this clinical evaluation trial were evaluated by using the criteria of implant success presented at the 1998 Toronto Consensus Conference [34]. The evaluation criteria used were as follows: (1) The implant should not be an obstacle to installing a functional and aesthetic prosthesis that satisfies both patients and dentists; (2) the implant should not cause any pain, discomfort, paresthesia, or inflammation and apical radiographs should not show radiolucency around the implant; (3) implants not connected during clinical examinations should have no mobility; (4) marginal bone resorption up to one year after implant placement must be ≤1.6 mm and the average bone loss must be ≤0.2 mm per year after functioning for one year. The extent of bone loss was evaluated by using apical radiographs.

#### 2.3.2. Implant Marginal Bone Resorption

**Figure 4:**
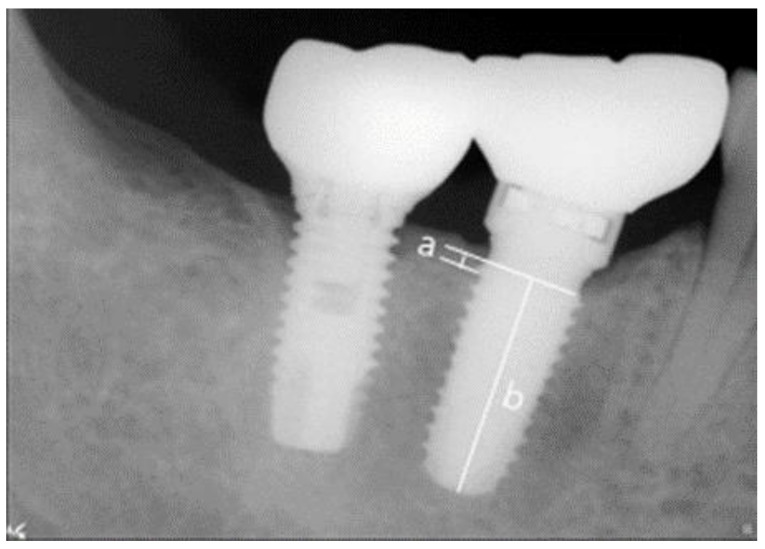
References used to measure actual marginal bone resorptions. (**a**) Marginal bone resorption and (**b**) implant fixture length.

Abutments were connected and, during periodic observations, digital intraoral radiographs were taken by using the parallel technique to evaluate surrounding bone resorption. After setting implant prostheses, mesial and distal bone levels were measured based on fixture-abutment junctions and vertical bone resorptions measured at 6 and 12 months after prosthesis placement were compared and analyzed. Radiographs were taken by using a Portable X-ray unit (Port II, Genoray, Seongnam, Korea) and measurements were obtained by using the PACS software (Digi-X, Hanjin Digi-X Co., Seoul, Korea). The means and standard deviations were calculated after scale correction based on known fixture lengths (Figure 4) [29,35].

#### 2.3.3. Probing Depth

Mean probing depths were measured on the mesiodistal and labio-lingual sides of implants at positions parallel to the long axes of the implant by using a periodontal probe (Merrit-B; Hu-Friedy, Chicago, IL, USA) at the scheduled 6 month and 12 month checkup visits [36].

#### 2.3.4. Modified Plaque Index (mPI)

The amount of plaque attached to implant surfaces was assessed at 6 months and 12 months after prosthesis placement and scored from 0 to 3 (Table 2) by using the criteria suggested by Mombelli et al. [37].

#### 2.3.5. Modified Sulcus Bleeding Index (mBI)

Degrees of bleeding after probing were assessed by using a periodontal probe and scored from 0 to 3 (Table 3) using the criteria suggested by Mombelli et al. [37].

#### 2.3.6. Complications

After prosthesis placement, the complications were investigated and types and frequencies of complications were recorded. Complications for all prosthesis-related problems were examined. All complications listed in the chart were recorded during regular and final checkups. Complications that occurred multiple times for one implant were counted as individual complications.

### 2.4. Statistical Analysis

The formula used for calculating the number of subjects required for the analysis is as follows.

n = 2 × (Z_1 − α_ + Z_1 − β_)^2^ × σ^2^/(μ_1_ − μ_2_ − δ)^2^ = 2 × (1.96 + 0.84)^2^ × 1.1^2^/1^2^ = 18.9728 ≒ 19

Z_1 − α,_ Z_1 − β_ was the quantile value from the normal distribution. Based on the findings of a previous non-inferiority margin, (δ) was set at 1.0, significance level (α) at 5%, power (β) at 80%, and standard deviation (σ) at 1.1 [29].

In order to compare marginal bone resorptions and probing depths in the MLF and MLA groups, the independent *T*-test was performed on normally distributed variables and the Mann–Whitney U test was used when variables were non-normally distributed. The chi-square test was performed to compare the values of group mPI and mBI. The analysis was performed by using SPSS ver. 25.0 (SPSS Inc., Chicago, IL, USA) and statistical significance was accepted for *p* values < 0.05.

## 3. Results

### 3.1. Cumulative Implant Survival Rates

During one year of follow-up after prosthesis placement, no implant failed in either group. All implants functioned normally without clinical mobility (Table 4).

### 3.2. Implant Marginal Bone Resorption

Mean and standard deviations of implant marginal bone resorptions are presented in Table 5. In the MLF group, implant marginal bone resorptions at 6 and 12 months after prosthesis placement were 0.43 ± 0.24 and 0.44 ± 0.25 mm, respectively, and the corresponding values were 0.31 ± 0.24 and 0.33 ± 0.34 mm in the MLA group. No significant intergroup or intragroup differences were found (both *p* > 0.05).

### 3.3. Probing Depth

The mean and standard deviations of probing depths are presented in Table 6. In the MLF group, mean probe depths at 6 and 12 months were 2.12 ± 0.66 and 2.22 ± 0.56 mm, respectively, and corresponding values in the MLA group were 2.37 ± 0.53 and 2.49 ± 0.45. No significant intergroup or intragroup difference was found at 6 or 12 months (both *p* > 0.05).

### 3.4. Modified Plaque Index (*mPI*)

In both groups, a score of 0 was obtained most frequently. At the 6 month checkups, mean mPI scores were non-significantly different in the two groups (*p* > 0.05), but the mean mPI score was significantly lower in the MLF group at 12 months (*p* < 0.05). No significant intragroup differences were observed at 6 or 12 months (*p* > 0.05) (Table 7).

### 3.5. Modified Sulcus Bleeding Index (*mBI*)

In both groups, a score of 0 was observed most frequently. No significant intergroup or intragroup difference was observed between mBI values at 6 or 12 months (*p* > 0.05) (Table 8).

### 3.6. Complications

More complications were observed at the 6 month check-ups than at 12-month checkups (Table 9). At 6 months in the MLF group, food impaction was observed in 15.8%, detached prosthesis in 5.3%, and gingival swelling in 5.3%. In the MLA group, complication rates included screw loosening in 5.3%, detached prosthesis in 5.3%, and food impaction in 5.3%. In the MLF group at 12 months, a detached prosthesis was observed in 5.3% and graft food impaction in 10.5%. No complication was observed in the MLA group at 12 months.

## 4. Discussion

Screw-retained prostheses are widely used in clinical practice but can cause complications such as screw loosening, screw fracture, and abutment fracture [38]. Cement-retained prostheses also have some limitations, such as infection and marginal bone resorption by residual cement. In addition, the prosthetic margin may be positioned below soft tissue due to aesthetic requirements and the removal of residual cement in such cases may damage the soft tissue around implants [39].

Various methods have been devised to overcome these shortcomings. A prosthesis retaining system using shape memory alloy was introduced and has been reported to be clinically successful. The Smileloc^®^ system (Rodo Medical, San Jose, CA, USA) is a typical example of this type of system and provides retrievability by using a shape memory nickel-titanium sleeve. Thus, cement-related peri-implant complications can be reduced and the structural integrity of the prosthesis can be improved by eliminating the occlusal screw access hole [18,40]. Another method to retain the prosthesis without cement uses a tight fit between a customized abutment and zirconia with a resin-coated inner surface. However, there is always a possibility of screw-related mechanical complications because these newly developed methods also rely on a screw to connect the implant fixture and abutment [41].

The ML system was developed to address these shortcomings. Initially, an abutment including an ML system was commercialized to facilitate application in existing implant systems. Several laboratory studies, case reports, and clinical studies have suggested the possible successful clinical application of the ML system [26,27,28]. However, in a 1 year prospective clinical study, the cumulative survival rate of implants in MLF was reported to be 100%, which indicates that changing the implant prosthesis connection method would not significantly affect implant survival [29].

A systematic review of clinical comparison of one piece versus two piece implants reported marginal bone resorption rates of 0.39 to 1.8 mm and 0.56 to 1.6 mm, respectively [42]. In the present study, the mean marginal bone resorptions at 6 months and 12 months after prosthesis placement in the MLF group were 0.43 ± 0.24 mm and 0.44 ± 0.25 mm, respectively, and the corresponding values in the MLA group were 0.31 ± 0.24 and 0.33 ± 0.34 mm, which concurs with the results of a systematic review of clinical comparisons of one piece versus two piece implants [42]. In the present study, no significant intergroup differences or changes were observed at 6 or 12 months after prosthesis placement.

An up to 6 month pilot clinical study of the Smileloc^®^ system found that the probing depth was less than 3 mm at all sites for all participants throughout the observation period [40]. In the present study, the mean probe depths at 6 and 12 months in the MLF group were 2.12 ± 0.66 and 2.22 ± 0.56 mm, respectively, and the corresponding values in the MLA group were 2.37 ± 0.53 and 2.49 ± 0.45. These results are similar to those obtained in the clinical study of the Smileloc^®^ system. In the present study, no significant intergroup difference or intragroup change was observed at 6 months or 12 months after prosthesis placement.

In a study that compared cement-retained and screw-retained prostheses, cement-retained prostheses generally showed more plaque deposits and bleeding [43]. Periodontal indices of cement-retained prostheses remained high during a three-year observation period, while the screw-retained prostheses showed a stable plaque deposit rate and bleeding level after 6 months. In the present study, a comparison of soft tissue statuses in the two groups using mPI and mBI measurements showed that the MLF group had the better periodontal condition at 12 months, but no significant differences were observed between the two groups at 6 months. This result indicates that implants in the MLF group were more compatible with soft tissue around implants. One piece implants have the advantage of fewer biological complications because no micro gap exists and no micromotion occurs at the connection between the abutment and fixture [30]. One piece implants in the MLF group were structurally simpler than the two piece implants in the MLA group and the MLF implants had no micro-gap; thus, it seems that this design has a beneficial effect on soft tissue.

Screw loosening, porcelain fracture, resin dropout from an access hole, and abutment fracture have been reported to be major complications of screw-retained prostheses and screw loosening, porcelain fracture, abutment fracture, and decementation of prostheses have been reported as complications of cement-retained prosthesis [38,44,45,46,47,48]. We also observed screw loosening, gingival swelling, prosthesis detachment, and food impaction. In the MLF group, food impaction was the main complication and, in the MLA group, the complications (in decreasing order) were screw loosening, prosthesis detachment, and food impaction at 6 months. Since the prostheses used in the MLF group had a screw-free design, screw loosening was not an issue, which is an advantage shared with one piece implants without a screw component [30].

The main complication in the MLF group was food impaction, which is affected by occlusal relationships, implant location in the arch, marginal ridge height difference versus proximal teeth, tightness of fit with proximal teeth, and the mobility of proximal teeth rather than the method used to connect the fixture and prosthesis [49]. It could be reasonably considered that food impaction as a complication can appear regardless of the prosthetic connection method used for most common implant prostheses. In order to reduce food impaction, a healthy proximal tooth without mobility or caries and a prosthesis with an appropriate contact point and tightness is required [50]. In this study, prosthesis detachment was observed in all groups. We suggest that additional studies with more samples be undertaken and that retention control should be considered.

Screw loosening in the MLA group occurred in 5.3% of patients 12 months after prosthetic cementation. The micro-locking abutment is manufactured to be compatible with the morse taper fixtures of various companies, but slight displacements may occur at the implant–abutment connection [29]. Therefore, the use of a micro-locking one-piece fixture is considered an attractive alternative.

In this prospective clinical study, MLF and MLA groups were compared over one year and the study failed to reveal significant differences between the clinical indices of the micro-locking one-piece fixtures and micro locking abutments. Therefore, we suggest that long-term clinical studies be conducted to identify complications not found in this study and to establish the clinical efficacy of the micro-locking one-piece fixture.

## 5. Conclusions

In this study, the clinical usefulness of the newly developed micro-locking one-piece fixture was confirmed by comparing it with the micro-locking abutment by clinical observation for one year after prosthesis placement. Within the limits of this study, we suggest that the micro-locking one piece fixture might be utilized as a new implant prosthesis retaining method that is free of the problem of screw loosening. Furthermore, our results indicate that this system provides a predictable treatment method. Larger and long-term clinical studies will be required to confirm our findings.

## Figures and Tables

**Table 1:** Distribution of implant placements in each group.

Group	Location	Anterior	Premolar	Molar	Total (*n*)
MLF	Maxilla	0	7	1	8
	Mandible	0	4	7	11
MLA	Maxilla	1	6	4	11
	Mandible	0	2	6	8

MLF = micro-locking one-piece fixture, MLA = micro-locking abutment.

**Table 2:** Assessment of plaque accumulation by using modified plaque indices.

Score = 0	No detection of plaque.
Score = 1	Plaque only recognized by running a probe across the smooth marginal surface of the implant. Implants covered by titanium spray in this area were scored as 1.
Score = 2	Plaque observed by naked eye.
Score = 3	Abundant soft matter.

**Table 3:** Assessment of bleeding tendency by using modified sulcus bleeding indices.

Score = 0	No bleeding when a periodontal probe is passed along the gingivalmargin adjacent to an implant.
Score = 1	Isolated bleeding spots visible.
Score = 2	Blood forms a confluent red line on the margin.
Score = 3	Heavy or profuse bleeding.

**Table 4:** Cumulative survival rates of implants.

Group		Implants	Failed Implants	CSR (%)
MLF	6 months	19	-	100
	12 months	19	-	100
MLA	6 months	19	-	100
	12 months	19	-	100

CSR = cumulative survival rates of implants, MLF = micro-locking one-piece fixture, MLA = micro-locking abutment.

**Table 5:** Mean values and standard deviations of marginal bone resorption.

Group	Marginal Bone Resorption (mm)		*p* Value
	6 Months	12 Months	
MLF MLA	0.43 ± 0.24	0.44 ± 0.25	0.872
	0.31 ± 0.24	0.33 ± 0.34	0.661
*p* value	0.096	0.060	

MLF = micro-locking one-piece fixture, MLA = micro-locking abutment.

**Table 6:** Mean values and standard deviations of probing depths.

Group	Probing Depth (mm)		*p* Value
	6 Months	12 Months	
MLF MLA	2.12 ± 0.66	2.22 ± 0.56	0.629
	2.37 ± 0.53	2.49 ± 0.45	0.274
*p* value	0.205	0.054	

MLF = micro-locking one-piece fixture, MLA = micro-locking abutment.

**Table 7:** Modified plaque index (mPI).

	Score	Occurrence Rate (%)		*p* Value
		MLF	MLA	
6 months	0	78.9	57.9	0.330
	1	15.8	36.8	
	2	5.3	5.3	
	3	-		
12 months	0	84.2	51.6	0.036 *
	1	15.8	47.4	
	2	-	-	
	3	-	-	
*p*-value		0.597	0.523	

MLF = micro-locking one-piece fixture, MLA = micro-locking abutment. (* *p* < 0.05).

**Table 8:** Modified sulcus bleeding index (mBI).

	Score	Occurrence Rate (%)		*p* Value
		MLF	MLA	
6 months	0	73.7	84.2	0.528
	1	21.1	15.8	
	2	5.3	-	
	3	-	-	
12 months	0	68.4	73.7	0.487
	1	31.6	21.1	
	2	-	5.3	
	3	-	-	
*p* value		0.487	0.528	

MLF = micro-locking one-piece fixture, MLA = micro-locking abutment.

**Table 9:** Detected clinical complications at 6 and 12 months.

Complications	6 Months		12 Months		Total (*n*)
	MLF	MLA	MLF	MLA	
Screw loosening	0	1	0	0	1
Prosthesis detachment	1	1	1	0	3
Gingival swelling	1	0	0	0	1
Food impaction	3	1	2	0	5
Total (*n*)	5	3	3	0	10

MLF = micro-locking one-piece fixture, MLA = micro-locking abutment.

## Data Availability

Data sharing not applicable.
